# Effects of the Aqueous Extract from* Tabebuia roseoalba* and Phenolic Acids on Hyperuricemia and Inflammation

**DOI:** 10.1155/2017/2712108

**Published:** 2017-12-11

**Authors:** Zilma Schimith Ferraz-Filha, Fernanda Cristina Ferrari, Marcela Carolina de Paula Michel Araújo, Ana Catharina Fernandes P. F. Bernardes, Dênia Antunes Saúde-Guimarães

**Affiliations:** ^1^Laboratório de Plantas Medicinais, Escola de Farmácia, Universidade Federal de Ouro Preto, 35400-000 Ouro Preto, MG, Brazil; ^2^Departamento de Química, Instituto Federal de Minas Gerais, Campus Ouro Preto, 35400-000 Ouro Preto, MG, Brazil

## Abstract

*Tabebuia* species (Bignoniaceae) have long been used in folk medicine as anti-inflammatory, antirheumatic, antimicrobial, and antitumor. The aim of this study was to investigate if aqueous extract from the leaves (AEL) of* Tabebuia roseoalba* (Ridl.) Sandwith, Bignoniaceae, and its constituents could be useful to decrease serum uric acid levels and restrain the gout inflammatory process. HPLC analysis identified caffeic acid and chlorogenic acid in AEL. Antihyperuricemic effects and inhibition of liver XOD (xanthine oxidoreductase) by AEL and identified compounds were evaluated in hyperuricemic mice. Anti-inflammatory activity was evaluated on MSU (monosodium urate) crystal-induced paw edema. In addition, AEL antioxidant activity* in vitro* was evaluated. AEL, caffeic, and chlorogenic acids were able to reduce serum uric acid levels in hyperuricemic mice probably through inhibition of liver xanthine oxidase activity and significantly decreased the paw edema induced by MSU crystals. AEL showed significant antioxidant activity in all evaluated assays. The results show that the AEL of* Tabebuia roseoalba* can be a promising agent for treatment for gout and inflammatory diseases. We suggest that caffeic and chlorogenic acids may be responsible for the activities demonstrated by the species.

## 1. Introduction

More than 200 years ago, hyperuricemia is known to be causally involved in the pathogenesis of gout, a painful inflammatory arthritis induced by the deposition of monosodium urate (MSU) crystals in synovial fluid and other tissues [1]. Recent studies suggest that the prevalence and incidence of gout have increased in the last decades affecting approximately 1-2% of adult males in occidental countries [2]. Currently, hyperuricemia has become a focus of research because of its association with a number of clinical disorders other than gout, including hypertension, atherosclerosis, cardiovascular disease, and chronic kidney disease [3]. The final product of purine metabolism in human is uric acid and it is generated by the action of xanthine oxidase (XO) enzyme, which catalyzes the conversion of hypoxanthine to xanthine and of xanthine to uric acid [3]. Hyperuricemia occurs due a decrease in uric acid excretion, an overproduction of uric acid, or an excessive intake of purines [2]. Current acute gout treatment focuses on controlling pain and inflammation. Drugs such as colchicine, NSAIDs (nonsteroidal anti-inflammatory drugs), and analgesics are part of the therapeutic regime for gout. Once the acute crisis is controlled, drugs that reduce uric acid are prescribed. These drugs include allopurinol, which inhibit uric acid production, benzbromarone and probenecid, which increase its renal excretion [2, 4]. However, these drugs are known to cause severe gastrointestinal, renal, and cardiovascular adverse effects [5]. Therefore, the development of a new effective and safety antihyperuricemic and anti-inflammatory drug could be useful in gout therapy and is highly justified.

Bignoniaceae family includes 120 genus with 800 species mainly distributed within tropical and neotropical regions of America, Asia, and Africa [6].* Tabebuia* is the largest and the most important genus of the Bignoniaceae family, with approximately 100 woody species. In Brazil, they are popularly known as “ipê,” “pau-d'arco,” and “ipeúva.”* Tabebuia* species are used in folk medicine as anti-inflammatory, antifungal, antiparasitic, anticancer, analgesic, and antibacterial agents, which have been experimentally evaluated by several research groups [7–9]. In previous study, we evaluable effects of the ethanolic extract from leaves of* T. roseoalba* and of its constituents on hyperuricemia and inflammation [10]. Therefore, in this study, we aimed to investigate the pharmacological roles of aqueous extract from the leaves (AEL) of* T. roseoalba* and its constituents on* in vivo* gout models (*in vivo* anti-inflammatory, antihyperuricemic, and liver XOD inhibitory activities). In addition, antioxidant activity of AEL was evaluated too.

## 2. Materials and Methods

### 2.1. Chemicals

Xanthine, indomethacin, uric acid, linoleic acid and potassium persulfate (≥99%), allopurinol, caffeic acid, chlorogenic acid, ABTS (azinobis 2,2-(3-ethylbenzothiazoline-6-sulphonic acid)), DPPH (2,2-diphenyl-1-picrylhydrazyl), quercetin and gallic acid (98%), potassium oxonate and trolox (6-hydroxy-2,5,7,8-tetramethylchroman-2-carboxylic acid) (97%), rutin (≥95%), *β*-carotene (≥93%), Tween 40, and Folin-Ciocalteu reagent were obtained from Sigma-Aldrich (USA). Ketamine and xylazine were obtained from Sespro Industria e Comercio Ltda (Brazil). Uric acid assay kit was purchased from Bioclin (Brazil). Monosodium urate (MSU) crystals were prepared according to a method as previously described [5]. All other chemicals were the highest analytic grade available.

### 2.2. Plant Material

Leaves of* Tabebuia roseoalba* (Ridl.) Sandwith, Bignoniaceae, were collected in Lagoa Santa, Minas Gerais, Brazil, in November 2011, with permission of the Chico Mendes Institute for the Preservation of Biodiversity (Instituto Chico Mendes de Conservação da Biodiversidade (ICMBio))/System of Authorization and Information in Biodiversity (Sistema de Autorização e Informação em Biodiversidade (SISBIO)) (license number 17021-5). The plant was identified by Dra. Viviane R. Scalon from the Institute of Exact and Biological Sciences of the Federal University of Ouro Preto (UFOP) (Instituto de Ciências Exatas e Biológicas da Universidade Federal de Ouro Preto). A voucher specimen (OUPR 28839) was deposited at the Herbarium of the Institute of Exact and Biological Sciences at UFOP, Ouro Preto, Brazil.

### 2.3. Preparation of Plant Extract

Plant material was air-dried at 37°C and reduced to powder. Leaves (70.0 g) were extracted by percolation with 5 L of water. Water was removed by lyophilization, yielding 7.1 g of dried crude aqueous extract from the leaves (AEL).

### 2.4. Characterization of the Extract by HPLC-UV/DAD Analysis

HPLC-UV/DAD analysis was performed using a Waters Liquid Chromatograph (model: Alliance 2695) equipped with a vacuum degasser, a quaternary pump, an autosampler, a diode array detector (DAD Waters 2996), and a reversed phase C18 column Shimadzu ODS (150 mm × 4.6 mm, 5 *μ*m). To assign compounds to the peaks the retention time and UV_*λ*max_ of standards eluted under the same conditions as the extract were used. The extract and the standards (caffeic and chlorogenic acids) were dissolved in methanol to yield a concentration of 1 mg/mL. The samples were filtered through 0.45 *μ*m Millex syringe filters. The volume injected was 25 *μ*L. Extract and standards were eluted in a gradient system with acidified water (0.1% formic acid) and methanol, starting with 100% of acidified water, reaching 5% of methanol in 2 minutes, 30% of methanol in 3 minutes, 75% of methanol in 50 minutes, and 80% of methanol in 52 minutes. The system was returned to the initial condition within 3 minutes. The flow rate was kept constant at 1.0 mL/min. The separation temperature was 25°C. The UV-DAD detector was set to record between 200 and 400 nm and UV spectra were recorded at 310 nm.

### 2.5. AEL Antioxidant Activity* In Vitro*

The methods and their description are adapted from our previous work [10].

#### 2.5.1. Measurement of Total Phenolic Content (TPC)

Folin-Ciocalteu adapted method was used [11]. Briefly, 500 *μ*L of AEL methanolic solution (100 *μ*g/mL) (blank: 500 *μ*L of methanol) was added to 500 *μ*L of Folin-Ciocalteu reagent and 500 *μ*L of 10% Na_2_CO_3_ solution. The absorbance was measured at 760 nm after 60 min of reaction, in the dark, at room temperature. Gallic acid was used as reference standard and TPC was expressed as mg of gallic acid (GAE) equivalent per gram of sample. Results were acquired using a calibration curve of freshly prepared gallic acid standard solutions (10–100 *μ*g/mL).

#### 2.5.2. Scavenging Ability of AEL Using DPPH Method

This assay using the DPPH was based on an adapted method [12]. AEL was dissolved in methanol to give the final concentrations of 10.0; 20.0; 30.0; 40.0; 50.0; and 100.0 *μ*g/mL. DPPH methanolic solution (1.5 mL, 100.0 *μ*g/mL) was added to 750 *μ*L of each AEL solution and kept at 32°C for 30 minutes. The absorbance of the reaction mixture was measured at 517 nm. Quercetin and trolox were used as positive controls. Results were expressed as percentage of antioxidant activity (AA) and calculated as follows: AA (%) = [1 − (Abs. sample − Abs. control/Abs. blank) × 100].

#### 2.5.3. Scavenging Ability of AEL Using ABTS Method

The method was carried out according on Rufino et al. [13]. AEL was dissolved in methanol to give the final concentrations of 1.0; 10.0; 20.0; 30.0; 40.0; and 50.0 *μ*g/mL. ABTS radical solution (3.0 mL) was added to 30 *μ*L of extract solution. The absorbance was measured at 734 nm after 6 minutes of incubation. Trolox and quercetin were used as positive controls. The ability of scavenging ABTS was expressed as percentage of antioxidant activity (AA) and as Trolox Equivalent Antioxidant Capacity (TEAC) and calculated as

(1) AA%=1−Abs.  sample−Abs.  controlAbs.  blank×100TEAC=mg  of  the  extract  equivalent  to 1000 μM  of  Trolox.

#### 2.5.4. *β*-Carotene Bleaching Assay

The assay was carried out according on Duarte-Almeida et al. [14] and Rufino et al. [15] with modifications. AEL was dissolved in methanol to give the final concentrations of 1.0; 10.0; 20.0; 30.0; 40.0; and 50.0 *μ*g/mL. *β*-Carotene chloroform solution (13.0 *μ*L, 20 mg/mL), linoleic acid (14.0 *μ*L), Tween 20 (183 *μ*L), and chloroform (0.5 mL) were mixed and chloroform was removed using a rotatory evaporator at room temperature. Then, 50 mL of distilled water previously oxygenated by bubbling was added to obtain an emulsion with an absorbance value of 0.60 ± 0.10 at 470 nm. Then, an aliquot of 2.5 mL of this emulsion was added to tubes containing extract solution (0.2 mL) placed in water bath at 40°C and the oxidation was monitored at 470 nm at the initial time and at 30 minutes intervals for 120 minutes. Trolox and quercetin were used as positive controls. The decrease in absorbance of samples (Abs. sample) was correlated with the absorbance of the system (Abs. system) in order to obtain the percentage of antioxidant activity or inhibition of oxidation (AA) by the equation:

(2) $$\begin{array}{c} \text{AA}\left(\;\%\;\right)=\left[\;1-\frac{\left(\Delta \text{Abs. sample}\right)}{\left(\Delta \text{Abs. system}\right)}\;\right]\times 100, \end{array}$$

where ΔAbs. sample is Abs. sample time 0 − Abs. sample time 120 minutes and ΔAbs. system is Abs. system time 0 − Abs. system time 120 minutes.

### 2.6. Animals Used

The Federal University of Ouro Preto (Ouro Preto, Minas Gerais, Brazil) provided male albino Swiss mice (25–30 g). Animals were divided into experimental groups (*n* = 6), housed in plastic cages, and maintained on a 12-h/12-h dark/light cycle with access to water and food ad libitum. All experimental procedures were approved by the Ethical Committee of the Federal University Ouro Preto, Brazil (protocol number: 2012/67 and 2015/06), and are compliant with the Guide for the Care and Use of Laboratory Animals, published by the Manual of Care and Procedures with Laboratory Animals of the Production and Experimentation Laboratory of Faculty of Pharmaceutical Sciences and Institute of Chemistry of the University of São Paulo [16].

### 2.7. Antihyperuricemic Activity

#### 2.7.1. Animal Model of Hyperuricemia in Mice

**Scheme 1:**
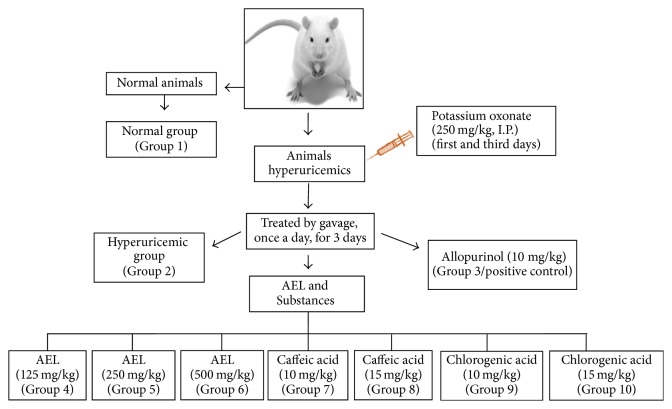
Experimental design for the antihyperuricemic activity assay. On the third day, 1 h after the final drug administration, mice were anesthetized with ketamine and xylazine. Then (1) blood was collected from abdominal aorta. Serum was stored at −20°C until assayed for uric acid quantification. (2) The liver was removed, washed in 0.9% saline, and stored at −80°C.

Antihyperuricemic activity of AEL, caffeic, and chlorogenic acids was evaluated as described elsewhere and their description is adapted from our previous work [10, 17–19]. Substances were solubilized in vehicle [DMSO : Tween 80 : water (1 : 1 : 8)]. AEL was solubilized in water. Mice were divided into 10 experimental groups (*n* = 6). Animals from groups 1 and 2 (normal and hyperuricemic control groups) received only vehicle, by gavage. Mice from group 3 (positive control) were treated by gavage with allopurinol (10 mg/kg body weight). Animals from groups 4 to 6 were treated by gavage with AEL (125, 250, and 500 mg/kg body weight). Mice from groups 7, 8, 9, and 10 were treated by gavage with caffeic (10 and 15 mg/kg body weight) and chlorogenic acids (10 and 15 mg/kg body weight), respectively. Potassium oxonate, dissolved in 0.9% saline solution (250 mg/kg, IP), was administrated to the animals, except group 1, on the first and third days of the experiment, 1 h before oral administration of tested compounds. Oral treatments with extract, caffeic and chlorogenic acids, allopurinol, and vehicle were given by gavage, once a day, for 3 days. On the third day, mice were anesthetized with ketamine and xylazine (100 and 20 mg/kg, resp.), 1 h after the final drug administration. Then, blood was collected from abdominal aorta, allowed to clot for approximately 1 h at room temperature, and centrifuged at 3,000 ×g for 10 min. Serum was stored at −20°C until assayed for uric acid quantification. The liver was removed, washed in 0.9% saline, and stored at −80°C (Scheme 1).

#### 2.7.2. Uric Acid Assay

Serum uric acid was determined using a standard diagnostic kit (Bioclin, Brazil) according to manufacturer's instructions.

#### 2.7.3. Assay of XOD Activity

Enzyme extraction was performed as described elsewhere [10, 18, 19]. Briefly, livers were homogenized with 5 mL of 50 mM sodium phosphate buffer (pH 7.4). Then, the homogenate was centrifuged at 3,000 ×g for 10 min at 4°C. The lipid layer was carefully removed and supernatant was further centrifuged at 10,000 ×g for 60 min at 4°C. The final supernatant (liver homogenate) was used for enzyme assay. The reaction mixture was made with 5,000 *μ*L of phosphate buffer (50 mM, pH 7.4) containing potassium oxonate (1 mM), in order to avoid oxidation of the formed uric acid into allantoin and 100 *μ*L of liver homogenate. A preincubation of 15 min at 37°C was performed and the reaction was initiated by the addition of 1,200 *μ*L of xanthine (250 mM). The reaction was stopped after 0 and 30 min by adding 500 *μ*L of HCl (0.6 M) to the reaction medium. Then, solutions were centrifuged at 3,000 ×g for 5 min. The supernatant was separated and the absorbance measured at 295 nm using a Varian 50 Bio UV/VIS spectrophotometer. XOD activity was expressed as nanomoles of uric acid formed per minute per milligram of protein. Protein concentration was determined spectrophotometrically by the Bradford method (1976) [20] using bovine serum albumin (BSA) as standard. Each assay was performed in triplicate.

### 2.8. Anti-Inflammatory Activity

#### 2.8.1. MSU Crystal-Induced Inflammation in Mice

**Scheme 2:**
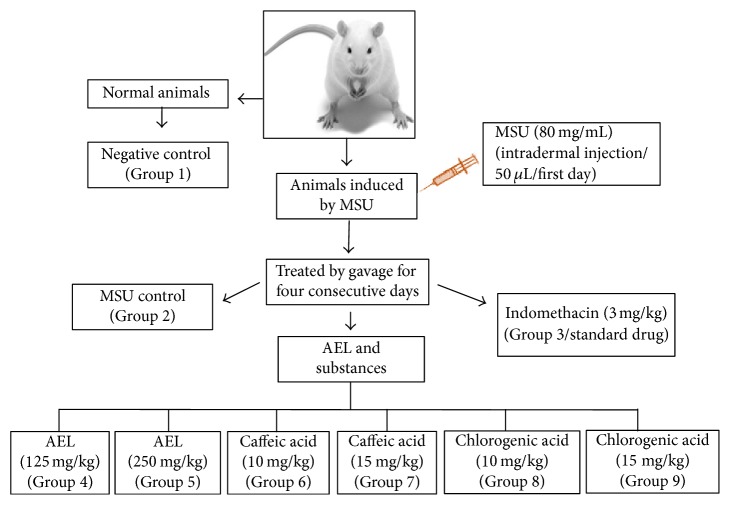
Experimental design for the anti-inflammatory activity assay. (1) Inflammatory swelling was expressed as thickness variation (∆). (2) Inflammation was quantified by measuring the thickness of the paw with caliper rule at 0, 4, 24, 48, and 72 h after MSU.

AEL, caffeic, and chlorogenic acids was evaluated by an experimental model of gout, as previously described [5, 10, 18, 19], with modifications. MSU crystals were suspended in 0.9% sterile saline solution (80 mg/mL) prior to use. On the first day of the experiment, the inflammation was induced by an intradermal injection of 50 *μ*L of MSU suspension in the mouse's right hind paw. The left paw was injected with saline (negative control). In both experiments (Figures 3 and 4) treatments were administrated by gavage, once a day, 1 h before MSU injection at the first day, and then repeated for three consecutive days at the same hour. Substances were solubilized in an appropriated vehicle [DMSO : Tween 80 : water (1 : 1 : 8)]. AEL was solubilized in water. Mice were divided into 9 groups (*n* = 6). At both experiments, mice from groups 1 and 2 were treated by gavage with vehicle and served as negative control and MSU-induced control, respectively. Animals from group 3 were treated by gavage with the standard drug indomethacin (3 mg/kg body weight). Animals from groups 4 and 5 were treated with AEL (125 and 250 mg/kg body weight). Animals from groups 6, 7, 8, and 9 were treated with caffeic (10 and 15 mg/kg body weight) and chlorogenic acids (10 and 15 mg/kg body weight), respectively. In both experiments, the inflammation was quantified by measuring the thickness variation (Δ) of the paw with a caliper rule (150 mm–6 in, Vonder, China) at 0, 4, 24, 48, and 72 h after MSU (Scheme 2).

### 2.9. Statistical Analysis

Results were expressed as mean values ± SEM. Experimental data were analyzed using GraphPad Prism 5.0 Software (Inc., San Diego, CA, USA). One-way analysis of variance (ANOVA) followed by Student's Newman-Keuls test was used to determine the significant differences between the groups. A *p* values of ≤0.05 was considered statistically significant.

## 3. Results

### 3.1. HPLC-UV/DAD Analysis

**Figure 1:**
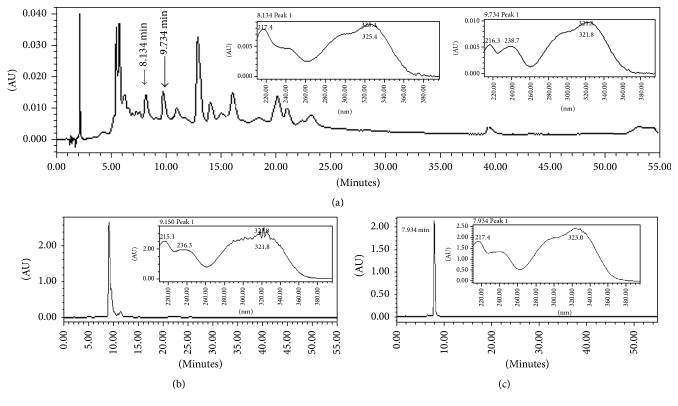
HPLC chromatograms of aqueous extract of leaves from* Tabebuia roseoalba* (AEL) (a) at 310 nm and UV spectra of identified and standards substances caffeic acid (b) and chlorogenic acid (c).

Analyses carried on HPLC-UV/DAD comparing retention time and UV spectra with standards confirmed the presence in AEL of caffeic (9.734 min, retention time) and chlorogenic acids (8.134 min, retention time) (Figure 1).

### 3.2. Antioxidant Activity

Aqueous extract from the leaves of* Tabebuia roseoalba* (Ridl.) Sandwith, Bignoniaceae (AEL), contains a low concentration of phenolic compounds (23.7 mg GAE/g). The evaluation of antioxidant activity indicates that AEL possesses an average antioxidant activity at the DPPH scavenging test, showing 51% of antioxidant activity at the concentration of 100 *μ*g/mL. When reacted with ABTS, the extract showed the highest scavenging activity (88% of 40 *μ*g/mL). AEL was also able to prevent more than 80% of *β*-carotene bleaching at the concentration of 40 *μ*g/mL (Table 1).

### 3.3. Antihyperuricemic Activity

**Figure 2:**
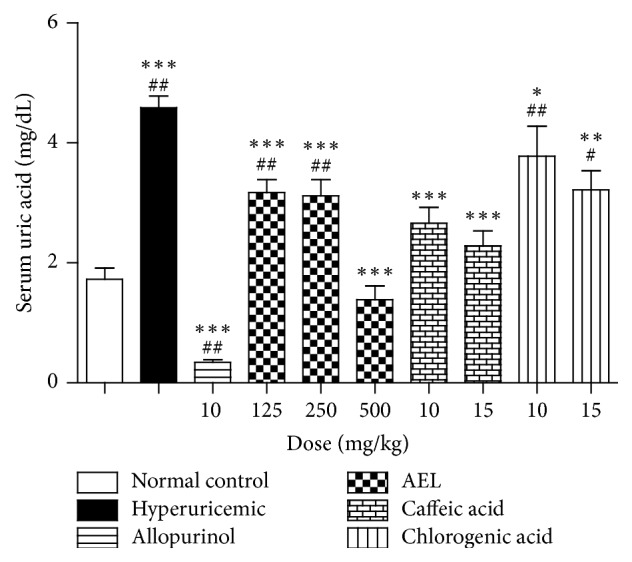
Effects of aqueous extract of leaves from* Tabebuia roseoalba* (AEL), caffeic acid, and chlorogenic acid on serum uric acid in hyperuricemic mice. Data representing serum levels of uric acid (mg/dL) of mean ± SEM. ^*∗*^*p* < 0,05, ^*∗∗*^*p* < 0,01, and ^*∗∗∗*^*p* < 0,001 compared with control group hyperuricemic; ^#^*p* < 0,05 and ^##^*p* < 0,001 compared to normal control (ANOVA followed by Student's Newman-Keuls test), *n* = 6.

The experimental model of potassium oxonate-induced hyperuricemia* in vivo* was used to evaluate the antihyperuricemic activity of AEL, caffeic, and chlorogenic acids on serum urate levels of hyperuricemic mice. Treatment with potassium oxonate caused hyperuricemia in mice, as indicated by a significant increase in serum uric acid levels when compared to the normal control group. Oral treatment with the reference drug allopurinol (10 mg/kg) was able to reduce serum urate levels of hyperuricemic mice to values lower than that found in normal control group. A three-day treatment with AEL (125, 250, and 500 mg/kg), caffeic, and chlorogenic acids (10 and 15 mg/kg) significantly reduced serum urate levels compared to hyperuricemic control group (Figure 2).

Treatments with AEL (125, 250, and 500 mg/kg), caffeic, and chlorogenic acids (10 and 15 mg/kg) were able to inhibit liver XOD activity when compared to hyperuricemic control group (Table 2).

The most expressive results were found for AEL (500 mg/kg) and caffeic acid (15 mg/kg), with inhibition liver XOD activity greater than 45% for both.

### 3.4. Anti-Inflammatory Activity

**Figure 3:**
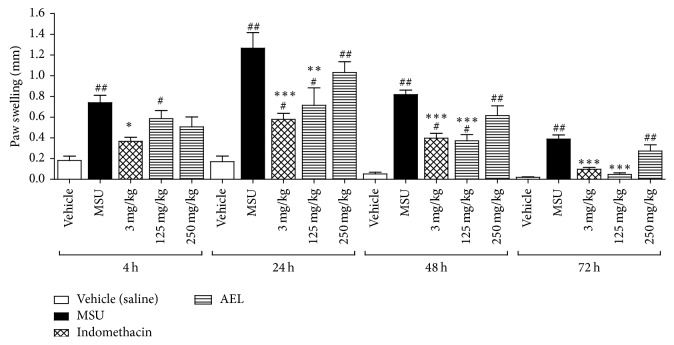
Effects of oral administration of aqueous extract of leaves from* Tabebuia roseoalba* (AEL) on MSU crystal-induced paw edema in mice. One-way ANOVA followed by Student's Newman-Keuls test was used for statistical significance. ^*∗*^*p* < 0.05, ^*∗∗*^*p* < 0.01, and ^*∗∗∗*^*p* < 0.001, compared with paw swelling after MSU crystals injection in vehicle-treated mice; ^#^*p* < 0.05 and ^##^*p* < 0.001, compared with paw swelling after saline injection in vehicle-treated mice.

**Figure 4:**
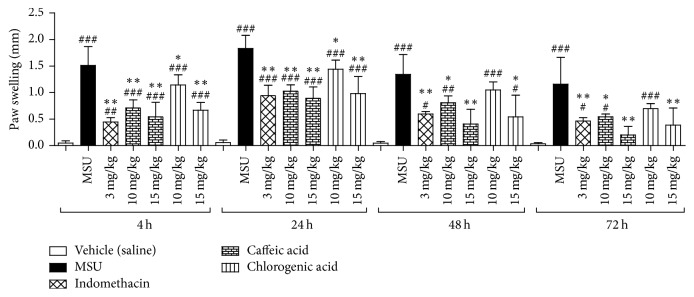
Effects of caffeic and chlorogenic acids on MSU crystal-induced paw edema in mice. One-way ANOVA followed by Student's Newman-Keuls test was used for statistical significance. ^*∗*^*p* < 0.05 and ^*∗∗*^*p* < 0.001, compared with paw swelling after MSU crystals injection in vehicle-treated mice; ^#^*p* < 0.05, ^##^*p* < 0.01, and ^###^*p* < 0.001, compared with paw swelling after saline injection in vehicle-treated mice.

Experimental model of gouty arthritis in mice was used in order to evaluate the anti-inflammatory activity of AEL, caffeic, and chlorogenic acid on MSU crystal-induced inflammation in mice. The injection of MSU crystals in the paw of mice produced a significant increase in paw thickness, as shown in Figures 3 and 4. In both experiments, indomethacin (3 mg/kg) caused significant anti-inflammatory effect at all evaluated times. Paw edema was found to be reduced in mice treated with AEL (125 mg/kg) at 24th, 48th, and 72nd h. These results did not show any statistical differences when compared to those of the group treated with indomethacin, an anti-inflammatory in the clinical use. Caffeic (10 and 15 mg/kg) and chlorogenic acids (15 mg/kg) caused a reduction on edema between the 4th and the 72nd h. These results did not show significant differences when compared to standard drug indomethacin at 24th, 48th, and 72nd h.

## 4. Discussion

Gout is a painful inflammatory arthritis caused by MSU crystals deposition in the joint fluid and adjacent tissues. The increasing on serum uric acid level is related to other conditions than gout, like cardiovascular diseases, obesity, hypertension, and resistance to insulin [1].

Plants and their extracts have been used in folk medicine to treat different diseases related to oxidative stress and inflammatory process. Some of these beneficial effects have been related to their antioxidant action. Oxidative stress has been recognized as a remarkable feature, which is frequently reported in the signalling cascade of inflammation and there are sufficient evidences suggesting its involvement in pathophysiology and complications of various diseases, including gout [21, 22].

The present study aimed to investigate the effects of the aqueous extract from the leaves of* T. roseoalba* (AEL) and its constituents on inflammation and hyperuricemia using animal models. Antioxidant activity of AEL was evaluated too.

The antihyperuricemic activity showed by AEL was followed by liver XOD inhibition activity and this effect was dose-dependent. The hypouricemic effect of caffeic and chlorogenic acids was related to liver XOD inhibition too. Thus, the mechanism by which treatment with AEL, caffeic, and chlorogenic acids reduced serum uric acid levels is related to the inhibition of liver XOD enzyme. A previous study carried out with the ethanolic extract from the leaves of* T. roseoalba* (EEL) (125, 250, and 500 mg/kg) also demonstrated an antihyperuricemic activity through inhibition of liver XOD [10]. The results suggested that the substances identified in EEL, stigmasterol, *β*-amyrin, and rutin contributed to EEL antihyperuricemic activity [10]. Thus, both aqueous and ethanolic extracts of the leaves from* T. roseoalba* exerted their hypouricemic effects due to inhibition of XOD.

Inflammation is the initial response of body to tissue damage caused by chemical, mechanical, or microbial stimuli. The cell activation by MSU microcrystals is one of the central features of acute gout. The main cells involved in the inflammatory response are monocytes/macrophages, polymorphonuclear leucocytes, and endothelial cells. When these cells become activated, they aggregate and infiltrate tissues where they undergo a respiratory burst, increasing their oxygen use and production of reactive oxygen species (ROS), cytokines as tumor necrosis fator (TNF-*α*), interleukins such as IL-1, IL-6, and IL-8, and other mediators of inflammation. These events can initiate and also perpetuate inflammatory cascades and cause subsequent tissue damage [22, 23]. It is known that drugs with anti-inflammatory and antihyperuricemic activities at the same time would be of interest for the treatment of gout [24].

Studies have suggested that rheumatic inflammatory diseases such as arthritis and atherosclerosis can be treated by the aqueous extract from the leaves of* Tabebuia avellanedae* (“ipê-roxo”) [9]. The anti-inflammatory activity found for the aqueous extract from the leaves of* T. roseoalba* (AEL) agrees with the activity reported by others species of* Tabebuia* genus, such as* T. avellanedae, T. impetiginosa*, and* T. chrysantha *[8]. Previous study showed a dose-dependent antiedematogenic effect of the ethanolic extract from the leaves of* T. roseoalba*. At the dose of 250 mg/kg, EEL was able to reduce the edema at 24th, 48th, and 72nd h [10]. The results indicated that the substances identified in EEL, rutin, and caffeic acid, stigmasterol, sitosterol, and *α*- and *β*-amyrins are probably related to the anti-inflammatory effect of the EEL [10].

In this study, same results were found to the lowest tested dose of AEL (125 mg/kg), demonstrating that the antiedematogenic effect of this extract was even better than the one found for EEL.

Caffeic and chlorogenic acids were able to inhibit XOD* in vitro*, with IC 50 values of 42.60 *μ*M and 56.21 *μ*M, respectively [25].

Study by Meng et al., 2014 [26], showed the hyperuricemic effect of chlorogenic acid in mice at doses of 50, 100, and 200 mg/kg in a dose-dependent manner and promoted the inhibition of hepatic XOD activity. In this same study, chlorogenic acid treatments (40, 80, and 160 mg/kg) reduced MSU crystal-induced inflammation in mice in a dose-dependent manner. In our study, chlorogenic acid showed antihyperuricemic activity, inhibited liver XOD activity, and promoted reduction of MSU-induced inflammation at a dose of 10 mg/kg [26].

Antihyperuricemic activity and effect on inhibition XOD hepatic* in vivo* of caffeic acid are shown for the first time in this study.

The presence of phenolic compounds in plant extracts is an indicative of a potential anti-inflammatory activity [27]. It is known that these kinds of phytochemicals have antioxidant properties and play a protective role in disorders related to oxidative stress, including pain and inflammation [22]. Indeed, previous studies demonstrated the antioxidant activity of caffeic acid [28–30] and chlorogenic acid [29, 31]. AEL has phenolic compounds such as chlorogenic and caffeic acids that can exert an important antioxidant activity. A single method can not fully evaluate the total antioxidant capacity, which varies depending on the adopted method and the system used as substrate. Different antioxidant compounds may act through different mechanisms [23]. Therefore, in this study, the antioxidant activity of AEL was evaluated using three different* in vitro* methods: DPPH and ABTS radical scavenging tests and the *β*-carotene bleaching assay.

Antioxidant activity measured by the *β*-carotene/linoleic acid system is very useful for the investigation of lipophilic antioxidants [14, 15]. Antioxidant activity of both lipophilic and hydrophilic constituents can be evaluated by ABTS radical method [13].

AEL showed a moderate antioxidant activity in DPPH assay and a high antioxidant activity in ABTS and *β*-carotene bleaching assay. The high activity of AEL as an ABTS radical scavenger is due to the capacity of this method to evaluate the antioxidant activity of both lipophilic and hydrophilic constituents [32]. The lipophilic nature of the *β*-carotene bleaching assay could be the determinant factor that explains the affinity of AEL antioxidant complex. Previous studies carried out by Genaro-Mattos et al. [30] proposed that the hydrophobicity of caffeic acid increases its antioxidant activity in a lipophilic environment. The authors indicated that the antioxidant effect of caffeic acid involves the prevention of initiation and propagation of lipid peroxidation by iron complexation and prevention of free hydroxyl radical formation. Therefore, caffeic and chlorogenic acids may be responsible for AEL antioxidant activity [30]. Antioxidant activity of AEL was in accordance with the one found for ethanolic extract from the leaves of* T. roseoalba*. In previous studies, EEL showed too a low concentration of phenolic compounds and higher antioxidant effects in the ABTS radical scavenger and *β*-carotene/linoleic acid system assays [10].

The antioxidant activity exerted by the AEL could explain, at least in part, the underlying mechanisms involved in its protective effects on the acute inflammation process [22]. Therefore, AEL anti-inflammatory activity can be partly attributed to the presence of substances with antioxidant properties, as caffeic and chlorogenic acid. Thus, these compounds may contribute to reduce the inflammatory process initiated by MSU crystals.

## 5. Conclusion

The aqueous extract from the leaves of* Tabebuia roseoalba* and caffeic and chlorogenic acids detected in this extract are potential agents for the treatment of inflammation, hyperuricemia, and gout.

## Figures and Tables

**Table 1:** Antioxidant activity and total phenolic content of aqueous extract of leaves from *Tabebuia roseoalba* (AEL) and positive controls using DPPH, ABTS, *β*-carotene, and Folin assays.

Sample	Tests										
	Folin mgGAE/g^*∗*^		DPPH			ABTS				*β*-Carotene	
AEL	23.65		AA% (100.0 *µ*g/mL)	IC_50_ (*µ*g/mL)		AA% (40.0 *µ*g/mL)	IC_50_ (*µ*g/mL)	ACET^*∗∗*^ (mean ± S.D.)		AA% (40.0 *µ*g/mL)	IC_50_ (*µ*g/mL)
			51	96.03 ± 3.93		88	20.34 ± 1.59	1.43 ± 0.08		81	12.24 ± 3.65

Positive controls			IC_50_ (*µ*g/mL)			IC_50_ (*µ*g/mL)		ACET^*∗∗*^ (mean ± S.D.)		IC_50_ (*µ*g/mL)	

Quercetin			8.40 ± 0.05			1.61 ± 0.04		0.10 ± 0.01		0.49 ± 0.04	
Trolox			15.62 ± 0.31			6.24 ± 0.16		—		1.86 ± 0.02	

^*∗*^mg/g gallic acid equivalent, values are means of triplicate determinations; ^*∗∗*^mg/mL of extract/control equivalent to 1000 *µ*M of trolox. EEL: ethanolic extract of leaves. GAE: gallic acid.

**Table 2:** Effects of aqueous extract of leaves from *Tabebuia roseoalba *(AEL), caffeic and chlorogenic acids on inhibition of xanthine oxidase activity in liver mice.

Treatment	Dose (mg/kg)	XOD activity (*U*/mg protein)	Inhibition (%)
Hyperuricemic control	—	15.82 ± 1.63	—

Allopurinol	10	3.83 ± 0.48^*∗∗*^	76

AEL	125	11.27 ± 2.61^*∗∗*^	29
	250	10.56 ± 2.97^*∗∗*^	33
	500	8.40 ± 2.70^*∗∗*^	46

Caffeic acid	10	11.28 ± 1.06^*∗*^	29
	15	8.58 ± 0.79^*∗∗*^	46

Chlorogenic acid	10	10.57 ± 0.71^*∗∗*^	33
	15	10.77 ± 1.12^*∗∗*^	32

Values were expressed as mean ± SEM of six animals. For statistical significance, one-way ANOVA was used followed by Student's Newman-Keuls test. ^*∗*^*p* < 0.01 and ^*∗∗*^*p* < 0.001, compared with hyperuricemic control group. *U* = nanomole uric acid/min.
